# Earlier antiretroviral initiation is independently associated with better arterial stiffness in children living with perinatally acquired HIV with sustained viral suppression in Mozambique

**DOI:** 10.4102/sajhivmed.v22i1.1282

**Published:** 2021-10-14

**Authors:** Igor S. Dobe, Ana O. Mocumbi, Noorjean Majid, Birhanu Ayele, Sara H. Browne, Steve Innes

**Affiliations:** 1 Division Non-Communicable Diseases, Instituto Nacional de Saúde, Maputo, Mozambique; 2 Department of Internal Medicine, Faculty of Medicine, University Eduardo Mondlane, Maputo, Mozambique; 3 Dream Center Sant’Egídeo, Maputo, Mozambique; 4 Division of Epidemiology and Biostatistics, Faculty of Medicine and Health Science, Stellenbosch University, Stellenbosch, South Africa; 5 Department of Medicine, Division of Infectious Diseases, University of California San Diego, San Diego, United States of America; 6 Department of Paediatrics and Child Health, Family Centre for Research with Ubuntu (FAMCRU), Stellenbosch University, Cape Town, South Africa; 7 Desmond Tutu HIV Centre, University of Cape Town, South Africa

**Keywords:** viral suppression, long-term ART, pulse wave velocity, large arterial elasticity, arteriosclerosis

## Abstract

**Background:**

Cardiovascular disease is a major driver of morbidity and mortality in adults living with HIV. The drivers of cardiovascular disease in children living with perinatally acquired HIV (PHIV) with sustained HIV viral suppression are unclear.

**Objectives:**

We explored the contribution of HIV-specific risk factors to arterial stiffness independently of traditional risk factors (metabolic syndrome [MetS]) in prepubertal children with PHIV with sustained viral suppression in a low-income country in Africa.

**Method:**

For this cross-sectional analysis, arterial stiffness was assessed by pulse wave velocity *z*-score (PWVz), measured using a Vicorder device. Metabolic syndrome components were measured. We retrospectively collected the antiretroviral therapy (ART) exposures, HIV stage, CD4 count and HIV viral load. A multivariate linear regression model was constructed for MetS components, retaining age and gender as obligatory variables. We then added HIV-related metrics to assess whether these had an independent or additive effect.

**Results:**

We studied 77 virally suppressed children with PHIV without evidence of cardiovascular disease (from medical history and physical examination). In the initial model, the PWVz was independently associated with each MetS component. The PWVz was higher in participants with proportionally greater visceral fat (waist/height ratio), elevated lipids (triglyceride/high-density lipoprotein ratio) and insulin resistance (log homeostatic model assessment [HOMA]). The addition of age at ART initiation increased the model *R*^2^ value from 0.36 to 0.43. In the resulting model, younger age at ART initiation was independently associated with a better PWVz (*P* < 0.001).

**Conclusion:**

Earlier ART initiation was independently associated with lower large artery stiffness. This effect was independent of the effect of elevated lipids, visceral fat and insulin resistance.

## Introduction

Effective antiretroviral therapy (ART) for HIV infection has increased life expectancy, with survival extending into decades.^1^ Cardiovascular disease is a leading co-morbidity and cause of mortality among adults living with HIV and is predicted to have a major impact on the quality and length of life in children and adolescents living with perinatally acquired HIV (PHIV).^2^

Cross-sectional evidence suggests an increased incidence of vascular disease in children with PHIV on ART.^3^ As the ART-treated paediatric populations in Africa expand, the importance of this issue is likely to increase considerably.^1^ Despite its importance, limited data exist regarding the HIV-specific drivers of increased cardiovascular disease risk in virally suppressed children on ART in Africa.^4^ This information is essential for determining the appropriate portion of limited public health resources that should be allocated to deal with this issue. We therefore designed a study to investigate the risk factors for cardiovascular disease in children with PHIV with sustained viral suppression in a low-income country in Africa.

## Research methods and design

### Study site

The study was conducted at Mavalane General Hospital and the Dream Centre Sant’Egídio, a public HIV treatment clinic in the centre of the city of Maputo, Mozambique.

### Study design and population

We conducted a cross-sectional analysis of a group of prepubertal children with PHIV on uninterrupted ART and with sustained viral suppression (HIV RNA polymerase chain reaction [PCR] viral load less than 40 copies/mL). The group comprised consecutive clinic attendees recruited between June and December 2017 with long-term undetectable HIV RNA PCR viral load at every time point in the previous 3 years (routinely measured 6-monthly) recorded on the paediatric HIV patient database. Eligible subjects were prepubertal children aged 6 to 12 years without evidence of cardiovascular disease (from a medical history and physical examination) and registered on the paediatric HIV patient databases at Mavalane General Hospital or the Dream Centre Sant’Egídio. Children younger than 6 years were deemed unable to cooperate with pulse wave velocity (PWV) testing, and children older than 12 years are unlikely to be prepubertal. The participants were all on a first-line ART regimen consisting of a nucleoside reverse transcriptase inhibitor backbone (mostly zidovudine) and a non-nucleoside reverse transcriptase inhibitor (mostly nevirapine). None had been exposed to a protease inhibitor.

### Data collation, procedures and definitions

We used a structured questionnaire for demographics, medical history and ART exposure history to supplement existing medical records. We measured weight using an Omron scale accurate to the nearest 0.1 kg, and height using a stadiometer accurate to the nearest 0.1 cm. All patients underwent a physical examination. The waist circumference was measured using a non-stretchable tape measure at the navel at the end of inspiration, and the waist-to-height ratio was calculated.^5^ We measured the resting blood pressure using a manual sphygmomanometer with the patient seated, and after 10 min of resting; three readings were taken and the average calculated. We measured the aortofemoral PWV using the oscillometric Vicorder device. Pulse wave velocity measurements were converted into *z*-scores for height and gender using published norms^6^ and standard methods.^7^ All anthropometric data were converted into *z*-scores using World Health Organization (WHO) reference norms (WHO AnthroPlus: https://www.who.int/growthref/tools/en/).^8^

We collected fasting insulin and glucose levels, homeostatic model assessment (HOMA) insulin resistance index calculated as fasting glucose (mmol/L) × insulin (mU/L)/22.5,^9^ total cholesterol, high-density lipoprotein (HDL) cholesterol, low-density lipoprotein (LDL) cholesterol, triglycerides, HIV RNA PCR viral load and CD4 count. An HIV RNA viral load assessment is routinely performed annually, and retrospective laboratory data were used to calculate the cumulative time with undetectable HIV RNA PCR viral load at every time point (defined as < 40 copies/mL).

### Statistical analysis

The data were captured using Epi Info version 7 and analysed using Statistical Package for the Social Sciences (SPSS) version 23. Continuous data were expressed as median (interquartile range) and categorical data as number (%). The participants’ demographics and clinical characteristics were analysed descriptively. The distribution of PWV *z*-score (PWVz) was confirmed to be normal using the Shapiro–Wilk test.

During the model building, the multivariable linear regression model for metabolic syndrome (MetS) components (Model 1) was constructed using a forward stepwise approach, retaining age and gender as obligatory variables. The starting MetS variables depicted visceral obesity, insulin resistance, dyslipidaemia (triglyceride/HDL ratio, total cholesterol and LDL cholesterol) and hypertension. Interaction variables were retained if significant (*P* < 0.05) and biologically plausible. Thereafter, HIV-related metrics were added to assess whether these had an independent or additive effect (Model 2).

## Results

In total there were 132 potentially eligible children registered on the clinic database. Of these, we recruited 77 children with PHIV without evidence of cardiovascular disease and with a suppressed viral load (median cumulative time with suppressed viral load: 4.1 years) and uninterrupted ART (described in Table 1). The anthropometric *z*-scores, CD4 count and WHO staging indicated that they were largely lean individuals with relatively normal CD4 counts (median 887 cells/µL) and minimal HIV disease. Overall, 13/77 (17%) had at least one metabolic abnormality; 2/77 (2.6%) had at least two metabolic abnormalities; 1/77 (1.3%) had three or more metabolic abnormalities; and 17/77 (22%) had an abnormally elevated PWV (PWVz > 2).

**TABLE 1:** Characteristics of the participants (*n* = 77).

Variable	Frequency			
	*n*	%	Median	IQR
**Age at study visit (years)**	-	-	10.0	8.6–12.0
**Calendar year of birth**				
<2007	21	27	-	-
2007–2009	44	57	-	-
>2009	12	16	-	-
**Ethnicity (Black)**	77	100	-	-
**Gender**				
Male	42	55	-	-
Female	35	45	-	-
*Z* **-scores**				
BMI	-	-	–0.1	–0.6–0.3
Weight for age	-	-	–1.0	–1.5–0.4
Height for age	-	-	–0.9	–1.7–0.1
Weight for height	-	-	1.0	–0.7–0.7
Waist circumference	-	-	0.0	–0.5–0.4
WHO HIV disease Stage 1	77	100	-	-
Age at ART initiation (years)	-	-	1.9	1.2–4.1
Cumulative ART exposure (years)	-	-	7.8	5.5–8.9
Cumulative time with suppressed HIV viral load, <40 copies/mL (years)	-	-	4.1	3.4–5.2
**NRTI backbone (other than lamivudine)**				
Zidovudine	71	92	-	-
Tenofovir	4	5	-	-
Abacavir	2	3	-	-
**Third ART drug**				
Nevirapine	76	98	-	-
Efavirenz	1	2	-	-
CD4 (cells/μL)	-	-	887	719–1034
CD4 (%)	-	-	34.3	30.5–38.1
RNA viral load, <40 copies/mL	77	100	-	-
**HOMA-IR**	-	-	0.7	0.4–1.0
Abnormal HOMA-IR (>2.5 for prepubertal)	3	4	-	-
Fasting glucose (mmol/L)	-	-	3.9	3.5–4.2
Abnormal fasting glucose (>5.5 mmol/L)	1	1	-	-
Total cholesterol (mmol/L)	-	-	3.7	3.2–4.3
Abnormal total cholesterol (>5.2 mmol/L)	3	4	-	-
HDL cholesterol (mmol/L)	-	-	1.6	1.4–2.3
Abnormal HDL (<0.9 mmol/L)	4	5	-	-
LDL cholesterol (mmol/L)	-	-	1.5	1.1–2.1
Abnormal LDL (>3.4 mmol/L)	1	1	-	-
Triglycerides (mmol/L)	-	-	0.9	0.6–0.9
Abnormal triglycerides (>1.7 mmol/L)	0	0	-	-
Fasting insulin (UI/mL)	-	-	4.5	2.3–6.0
Abnormal insulin (>23 UI/mL)	1	1	-	-
Patients with any metabolic abnormality	13	17	-	-
PWV *z*-score	-	-	-0.1	–1.5–2.4

IQR, interquartile range; BMI, body mass index; WHO, World Health Organization; LDL, low density lipoprotein; HDL, high-density lipoprotein; HOMA-IR, homeostatic model assessment insulin resistance index; ART, antiretroviral therapy; PWV, pulse wave velocity; NRTI, nucleoside reverse transcriptase inhibitor.

The PWV data were normally distributed (Shapiro–Wilk *P* = 0.34, skewness = 0.23, kurtosis = –0.54). The initial model for MetS components (Model 1, Table 2) revealed that the PWVz was independently associated with each component of the MetS, as expected: the PWVz was higher in participants with proportionally greater visceral fat (waist/height ratio), elevated lipids (triglyceride/HDL ratio, HDL) and resistance (log HOMA). The addition of age at ART initiation increased the *R*^2^ value of the model from 0.36 to 0.43. The resulting model (Model 2, Table 3) revealed that the PWVz was independently associated with younger age at ART initiation. In addition, there was a trend towards lower PWVz in those who had been virally suppressed for longer; however, this finding was not statistically significant (*P* = 0.08) (Table 4).

**TABLE 2:** Multivariable linear regression model with pulse wave velocity *z*-score as the outcome variable, optimised for metabolic syndrome components (Model 1).

Predictor	Coefficient estimate	Standard error	*t*-statistic	*P*
Constant	–50.8	17.6	–2.8849	0.006
Age at study visit	–0.005	0.2	–0.0236	0.98
Gender (male)	0.3	0.6	0.5445	0.59
log HOMA-IR	22.8	7.6	3.0129	0.004
Waist/height ratio	117.0	42.8	2.7306	0.008
HDL	15.3	7.0	2.1877	0.03
Triglyceride/HDL ratio	29.2	10.2	2.8573	0.006

HDL, high density lipoprotein; HOMA-IR, homeostatic model assessment insulin resistance index.

**TABLE 3:** Multivariable linear regression model with pulse-wave velocity *z*-score as the outcome variable, showing independent association with age at antiretroviral therapy initiation (Model 2).

Predictor	Coefficient estimate	Standard error	*t*-statistic	*P*
Constant	–35.6	16.7	–2.1316	0.04
Age at study visit	0.1	0.2	0.6041	0.55
Gender (male)	0.7	0.6	1.3198	0.19
log HOMA-IR	10.2	2.9	3.4893	0.001
Waist/height ratio	75.7	39.7	1.9015	0.06
HDL	15.8	6.6	2.3495	0.02
Triglyceride/HDL ratio	31.8	9.7	3.2673	0.002
Age at ART initiation (years)	–5.9	1.7	-3.4482	0.001

HDL, high density lipoprotein; HOMA-IR, homeostatic model assessment insulin resistance index; ART, antiretroviral therapy.

**TABLE 4:** Multivariable linear regression model with pulse wave velocity *z*-score as the outcome variable, showing a trend towards association with cumulative time with suppressed HIV.

Predictor	Coefficient estimate	Standard error	*t*-statistic	*P*
Constant	–39.0	16.5	–2.3641	0.02
Age at study visit	0.2	0.2	1.2216	0.23
Gender (male)	0.5	0.6	0.9351	0.35
log HOMA-IR	10.3	2.9	3.5988	0.0007
Waist/height ratio	86.8	39.5	2.2005	0.03
HDL	17.5	6.6	2.6601	0.01
Triglyceride/HDL ratio	31.8	9.5	3.338	0.002
Age at ART initiation (years)	–5.9	1.7	–3.4974	0.0009
Cumulative time with suppressed HIV viral load (years)	–0.3	0.2	–1.8091	0.08

HDL, high density lipoprotein; HOMA-IR, homeostatic model assessment insulin resistance index; ART, antiretroviral therapy.

## Discussion

Our data provide evidence that, after adjustment for traditional risk factors, younger age at ART initiation in prepubertal PHIV with a suppressed viral load and without previous cardiovascular disease is independently associated with better arterial stiffness, using PWV as the outcome measure. Age at ART initiation is a potent determinant of future health in PHIV.^10,11,12^

Extrapolating from adult data, the most likely mediator of excess vascular disease risk in children with PHIV with sustained viral suppression is HIV-associated chronic inflammation and immune activation. Abnormalities of vascular endothelial function, arterial elasticity and inflammatory biomarkers are reported in children with PHIV in high-resource settings after adjustment for traditional risk factors.^3^ This impact is over and above the impact of MetS on HIV-associated arterial stiffness seen in sub-Saharan African adults on ART.^13^ While the metabolic disturbances resulting from ART may contribute to HIV-associated arteriosclerosis in children with PHIV,^14,15,16^ this is likely far outweighed by the benefits of suppressing HIV replication.

That uncontrolled HIV viral replication is detrimental to arterial health is well known. Data from adult studies have indicated increased arterial stiffness in patients with uncontrolled HIV as compared with matched controls without HIV, which reverses with ART, suggesting that HIV viral replication is a potent driver.^17^ However, viral suppression does not entirely remove this risk: a recent study of PWV and carotid artery intima-media thickness in Ugandan adolescents living with PHIV showed evidence of structural vascular changes despite viral suppression,^18^ suggesting that factors other than viral replication contribute to arteriosclerosis in adolescents with PHIV. To investigate this, our study specifically examined children with PHIV with sustained viral suppression. Few previous studies have included African children with a suppressed viral load.^19^

In this study we collected data from children with PHIV with sustained viral suppression (without viral blips) on long-term ART in Africa and used PWV as the outcome measure. Pulse wave velocity, a well-described index of arterial stiffness, has a key role in detecting early structural arterial disease progression and is a powerful prospective predictor of cardiovascular events in at-risk groups and the general population.^20^ Measurement of PWV using oscillometry has been specifically validated against the tonometric SphygmoCor device in children.^21,22^ The PWV technique is reproducible, easy to use and portable, and it may be a valuable tool for screening young populations at increased risk of vascular disease. The Vicorder device in particular is technically simple, not operator dependent, has high precision and low inter- and intra-observer variability over repeated measurements, and is comfortable and acceptable to children, who typically fall asleep while the device is recording.^23^

Metabolic syndrome is relatively well defined for adults,^24^ with thresholds for each component that prospectively predict cardiovascular events in large population studies.^25^ However, equivalent studies in children and adolescents are lacking. As a result, there is no consensus on a definition for MetS in children, with the bulk of disagreement revolving around thresholds for the five components of MetS.^26^ In reality, these five components are each on a continuum, and rather than focusing on thresholds, the American Academy of Pediatrics recommends focusing on clustering of these components.^26^ In the current study, we analysed the MetS components as continuous variables and sought to adjust for them as a cluster (Model 1) before investigating the effect of HIV-specific metrics.

Premature cardiovascular disease will likely become an important contributor to morbidity and mortality in young adults with PHIV. Even with long-term viral suppression, their cardiovascular disease risk remains high. Early identification of those at risk and appropriate preventive intervention may be effective in reducing their cardiovascular disease burden. Pulse wave velocity may be a useful tool for surveillance of vascular health and may provide the basis for a screening algorithm for early identification of arterial disease in countries with a large population of children living with PHIV, such as Mozambique.

## Limitations

Second-hand tobacco smoke exposure could not be quantified. However, given the relatively low incidence of smoking in Mozambique, it is unlikely that the inclusion of this factor would have changed the findings.

This study lacked age-matched controls without HIV or children with PHIV without ART exposure. Fortunately, as a result of the ART roll-out in Mozambique, ART-naïve children living with perinatally acquired HIV are few, and this limitation was unavoidable.

Weight/height ratio was used in this study as the measure of visceral adiposity. It is possible that newer anthropometric indices such as the body roundness index may have produced a different result.^27^

Physical activity and diet were not estimated, and it is possible that inclusion of these metrics may have altered the findings.

All participants had sustained, long-standing viral suppression, which does not match the national children with PHIV subpopulation profile of Mozambique. However, the inclusion of poorly suppressed children on ART would have obstructed the aim of the study, which was to investigate the drivers of HIV-associated vascular disease in children who have successfully attained sustained viral suppression. The sample size was limited (*n* = 77). It is possible that a larger sample may have produced a different result.

## Conclusion

Earlier age at ART initiation was independently associated with improved large artery stiffness in childhood. This effect was independent of the effect of elevated visceral fat, lipids and insulin resistance. These findings suggest that earlier ART initiation may be protective against HIV-associated arterial stiffening in children living with PHIV.
